# Editorial: Microglia in neuroinflammation

**DOI:** 10.3389/fimmu.2023.1227095

**Published:** 2023-06-15

**Authors:** Pinar Ayata, Ido Amit, Carla M. Cuda

**Affiliations:** ^1^Neuroscience Initiative, Advanced Science Research Center, Graduate Program in Biology, The Graduate Center of The City University of New York, New York, NY, United States; ^2^Department of Systems Immunology, Weizmann Institute of Science, Rehovot, Israel; ^3^Division of Rheumatology, Department of Medicine, Feinberg School of Medicine, Northwestern University, Chicago, IL, United States

**Keywords:** microglia, pain, microbiome, Alzheimer’s disease, traumatic brain injury, diabetic retinopathy, glaucoma, retinal stroke

Microglia are a resident innate immune cell population of the central nervous system (CNS) derived from yolk sac erythro-myeloid progenitors that migrate to the developing brain prior to the formation of the blood-brain barrier (1). This critical cell population has gained considerable traction in the literature as it is considered a protective barrier from CNS damage and, yet, can also serve as a primary mediator of neuroinflammation (1). Under normal physiological conditions, microglia perform homeostatic functions, such as parenchymal surveillance, neurotrophic support, pathogen or debris removal, and maintenance of synaptic homeostasis and neuronal plasticity (1).

When CNS homeostasis is disrupted, microglia—as the brain’s primary innate immune cells—sense and respond to pathogen-, damage-, or neurodegeneration-associated molecular patterns (PAMPs, DAMPs, or NAMPs, respectively). In response, microglia may initiate “neuroinflammation” by modifying their activity, for example, by increasing phagocytic capacity or secreting pro-inflammatory cytokines, which under some pathological conditions, may promote leukocyte infiltration (2). Depending on the context, this response can have protective or harmful outcomes. It may also become rampant and drive neurodegenerative disease and other chronic CNS pathologies (2).

Yet, the notion that microglia, and the neuroinflammation induced by their aberrant activity, are strictly pathogenic players in CNS disease is rapidly evolving. There clearly exists a delicate circuitry of feedback mechanisms that maintain the balance of time-sensitive "regulators", be they secreted molecules, sensory receptors (e.g., TREM2) and signaling pathways, transcription, or epigenetic factors, designed to keep microglial function in check. The goal of this Research Topic is to further elucidate these time-sensitive regulatory circuits and how/when they impact microglial-mediated neuroinflammation.

Microglia are at the forefront of research into neuroinflammation associated with neurodegenerative diseases, including Alzheimer’s disease, and contributing articles to this Research Topic expound upon their role. Reid et al. uncovered that microglia and neurons under various stressors secreted calreticulin. This highly conserved chaperone typically resides in the endoplasmic reticulum but can be extracellularly released (3). Calreticulin has been found in human cerebrospinal fluid bound to amyloid beta (4). Reid et al. suggest that calreticulin may then serve as 1) an alarmin to recruit and activate microglia, 2) an extracellular chaperone to prevent amyloid-beta aggregation, and 3) a neuroprotectant by preventing amyloid-beta-induced, microglial-mediated neuronal loss. Karahan et al. examined the impact of the deletion of Abelson interactor family member 3 (*Abi3*), a candidate risk gene for Alzheimer’s disease enriched in microglia (4), in the early stages of the disease in the 5XFAD model of Alzheimer’s disease. Consistent with their prior investigations in the later stages of the disease (5), *Abi3* deletion resulted in increased amyloid-beta plaque load in conjunction with elevated levels of insoluble amyloid early in the disease course. However, Karahan et al. found that *Abi3* deletion in the earlier stages of the disease did not induce the concurrent increase in microgliosis or plaque-associated microglia identified in the later stages (5). These findings add to the growing body of literature suggesting that microglia-expressed risk genes possess distinct time-sensitive functions depending on the stage of Alzheimer’s disease.

Microglia as mediators of neuroinflammation associated with other chronic CNS pathologies are also gaining momentum. For instance, prolonged dysregulated activation of microglia following the initial insult in traumatic brain injury induces a secondary insult resulting in neuronal death and chronic neurodegeneration (6). Triggering receptor expressed on myeloid cells 2 (TREM2) is a type I transmembrane receptor found on microglia, and mutations in *TREM2* are associated with several neurodegenerative disorders, including Alzheimer’s disease (7). Katsumoto et al. showed that in the absence of TREM2, microglia fail to phagocytose degrading neurons, contributing to the accelerated neurodegeneration observed in the chronic phase of traumatic brain injury in a mouse model of tauopathy. The authors postulate that TREM2-deficient microglia in this system may instigate an incomplete blood-brain barrier (8) or may not develop into neuroprotective disease-associated microglia (DAM) (9) or white matter-associated microglia (WAM) (10) subsets, as TREM2 is required for these functions.

Other chronic CNS pathologies wherein microglia contribute to the disease include those affecting the eye, specifically diabetic retinopathy, glaucoma, and retinal stroke. Church et al. showed that the pharmacological depletion of microglia protected mice from retinal degeneration in a model of diabetic retinopathy. The authors took advantage of a mouse strain expressing a polymorphic variant of the *CX3CR1* gene identified in ~25% of the population that rendered the receptor less capable of binding its ligand, fractalkine, to mitigate microglia-mediated inflammation (11). Using this mouse strain, Church et al. showed that the protective effect of microglial depletion depends on a fully functioning CX3CR1 receptor. Reinehr et al. developed a new multifactorial glaucoma model, combining a model of spontaneous intraocular pressure with a model of autoimmune glaucoma, that developed more severe optic nerve degeneration and loss of retinal ganglion cells than its component models. Further, the authors showed that retinal microglia were more abundant and activated in the multifactorial glaucoma model. Zeng et al. attempted an *in vivo* assessment of the clinical significance of macrophage-like cells, including microglia, in retinal stroke. The authors identified increased density and morphological changes in the macrophage-like cell population indicative of aggregation and activation positively correlated with ischemia severity and disease duration. However, whether these macrophage-like cells are, indeed, microglia is yet to be determined.

In addition to original research submissions, our Research Topic focuses on emerging reviews of two highly relevant topics: 1) epigenetic regulation of pain-mediating cytokines/chemokines produced by microglia and 2) microglia as sensors of gut microbe-derived signals in the gut-brain axis. As microglia are implicated in promoting chronic pain upon activation through the production of cytokines/chemokines (12), Jiang et al. reviewed current literature focused on the epigenetic control of this activation-induced signaling mediator production by microglia. Specifically, Jiang et al. focused on histone modifications (acetylation, deacetylation, methylation) in microglia as targetable processes to control chronic pain. Focusing on extracellular mechanisms potentially influencing microglial function, the intestinal microbiome produces molecules that exert both local and systemic effects designed to maintain host health. However, an imbalance of gut microbiota composition has been linked to several psychiatric and neurologic disorders (13). D’Alessandro et al. reviewed current literature detailing the impact of microbiota-derived signals on microglial phenotype and function. These signals include those resulting from gut microbiota dysbiosis, vagal nerve stimulation, and small molecules produced by gut microbiota that make it into the systemic circulation, like short-chain fatty acids, lipopolysaccharide, and tryptophan metabolites. Indeed, D’Alessandro et al. highlighted that several of these signals might be targetable for modulating microglial contributions to brain disease.

We are only now scratching the surface regarding the contribution of microglia regulatory circuits to neurodegenerative disease and particularly in other CNS pathologies resulting from chronic neuroinflammation. Further, knowledge of the expansive regulatory mechanisms designed to keep microglial function in check at different stages of CNS pathophysiology is rapidly emerging. A more comprehensive mechanistic understanding of these time-sensitive regulatory circuits may allow us to modulate microglia activity and significantly advance the design of novel classes of microglia-based therapeutics. Contributions to this Research Topic have helped pave the way.

## Author contributions

PA, IA, and CMC drafted, edited, and revised the manuscript and agreed with the final version.
